# Hepatic Cannabinoid Signaling in the Regulation of Alcohol-Associated Liver Disease

**DOI:** 10.35946/arcr.v41.1.12

**Published:** 2021-09-23

**Authors:** Keungmo Yang, Sung Eun Choi, Won-Il Jeong

**Affiliations:** 1 Laboratory of Liver Research, Graduate School of Medical Science and Engineering, Korea Advanced Institute of Science and Technology, Daejeon 34141, Republic of Korea; 2 Biomedical Research Center, Korea Advanced Institute of Science and Technology, Daejeon 34141, Republic of Korea

**Keywords:** alcohol, CB1R, CB2R, cell communication, endocannabinoid, fatty liver, metabotropic glutamate receptor 5, xCT

## Abstract

**PURPOSE:**

The endocannabinoid system has emerged as a key regulatory signaling pathway in the pathophysiology of alcohol-associated liver disease (ALD). More than 30 years of research have established different roles of endocannabinoids and their receptors in various aspects of liver diseases, such as steatosis, inflammation, and fibrosis. However, pharmacological applications of the endocannabinoid system for the treatment of ALD have not been successful because of psychoactive side effects, despite some beneficial effects. Thus, a more delicate and detailed elucidation of the mechanism linking the endocannabinoid system and ALD may be of paramount significance in efforts to apply the system to the treatment of ALD.

**SEARCH METHODS:**

Three electronic databases (PubMed, MEDLINE, and Cochrane Library) were used for literature search from November 1988 to April 2021. Major keywords used for literature searches were “cannabinoid,” “cannabinoid receptor,” “ALD,” “steatosis,” and “fibrosis.”

**SEARCH RESULTS:**

According to the inclusion and exclusion criteria, the authors selected 47 eligible full-text articles out of 2,691 searched initially. Studies in the past 3 decades revealed the opposite effects of cannabinoid receptors CB1R and CB2R on steatosis, inflammation, and fibrosis in ALD.

**DISCUSSION AND CONCLUSIONS:**

This review summarizes the endocannabinoid signaling in the general physiology of the liver, the pathogenesis of ALD, and some of the potential therapeutic implications of cannabinoid-based treatments for ALD.

The prevalence of alcohol use disorder has been steadily rising around the world in recent years, and reducing the burden of alcohol-associated liver disease (ALD) caused by chronic alcohol consumption has become one of the most important global health issues.^1^,^2^ Excessive alcohol drinking (more than 40 g of pure alcohol per day) is closely associated with increased risk of all-cause mortality including chronic diseases, such as cancer, cardiovascular conditions, and neuronal diseases.^3^ ALD comprises a wide spectrum of liver injury including simple steatosis, steatohepatitis, liver cirrhosis, and hepatocellular carcinoma. The predominant cause of alcohol-associated liver disease, as evident by its name, is the persistent intake of alcohol, and yet the detailed mechanisms of ALD progression remain vague.^4^,^5^

ALD develops through complex signaling pathways in the liver.^6^ Chronic alcohol consumption not only elicits various responses by innate immune cells in the liver, but also contributes to the metabolic dysfunction of hepatocytes, such as the production of reactive oxygen species (ROS), the abnormal lipogenesis induced by endoplasmic reticulum stress or mitochondrial dysfunction, and the secretion of inflammatory cytokines.^6^ Apart from alcohol-induced effects, endogenous cannabinoids (endocannabinoids), which are lipid mediators, also were found to play an important role in provoking ethanol-induced hepatic steatosis.^7^ The study of endocannabinoids began with the discovery that delta 9-tetrahydrocannabinol (THC), the major psychoactive component of cannabis, binds to G-protein-coupled receptors and exhibits diverse biological effects in the brain depending on the types of functioning cells affected.^8^ Over the past 3 decades, mounting evidence has shown that in peripheral organs, endocannabinoids modulate the progression of various diseases including nonalcoholic fatty liver disease (NAFLD), liver fibrosis, and ALD.^9^ However, the underlying mechanisms and the specifics of the cannabinoid signaling are yet to be elucidated. The authors of this review recently reported, however, that alcoholic steatosis is promoted by endocannabinoid production in hepatic stellate cells (HSCs), which is mediated by metabotropic glutamate receptor 5 (mGluR5).^10^ This review explores cannabinoid signaling in regard to the general physiology of hepatic function, the pathogenesis of ALD, and the potential therapeutic implications for ALD.

## Methods and Results of the Literature Search

In-depth literature investigation was performed for this review article. Three online databases (PubMed, MEDLINE, and Cochrane Library) were used for literature search. The major search terms used were “cannabinoid,” “endocannabinoid,” “cannabinoid receptor,” “alcoholic liver disease,” “steatosis,” and “fibrosis.” Among the initial search results retrieved from the online databases, articles published later than April 2021 and duplicate articles were removed, and articles written in English were screened first. Then, the authors included peer-reviewed original articles on animal experiments or clinical trials and well-organized review articles relevant to the subject. Research articles without peer review, abstracts of conferences or posters, and articles with unclear research processes or insufficient data were excluded. As a result, 47 eligible full-text articles were selected from a total of 2,691 searched initially. All authors independently conducted literature searches using the same online databases, and then selected appropriate references according to the inclusion and exclusion criteria.

## Cannabinoid Signaling Systems and Hepatic Function

### Endocannabinoid System

Marijuana (*Cannabis sativa*) has been widely used for medical applications (e.g., analgesic, antiemetic, appetite stimulant) since its discovery in ancient times.^11^ Now it is better known to the public for its psychoactive effects such as euphoria, relaxation, increased awareness of sensation, and alteration of conscious perception.^12^ Among the 60 different ingredients of marijuana, early research focused on THC, a phytocannabinoid, as it has the strongest psychoactive property. Because of its highly lipophilic and hydrophobic properties, THC was believed to provoke its effects nonspecifically by perturbing the membrane phospholipids. This misunderstanding persisted until the revelation of two cannabinoid receptors: type 1 (CB1R) and type 2 (CB2R).^13^

In comparison to their expression in the central nervous system (CNS), such as in the brain and spine, CB1R and CB2R are relatively less distributed and work differently in peripheral organs.^14^,^15^ For instance, CB1R and its ligands have critical roles in the pathogenesis of chronic liver diseases, such as steatosis and liver fibrosis.^14^,^15^ Meanwhile, CB2R is mainly distributed in immune cells or hematopoietic organs, where it functions as a protective responder to specific pathological conditions, especially in liver fibrosis.^16^,^17^ Like marijuana, endocannabinoids generally consist of analogs of long-chain polyunsaturated fatty acids and have an arachidonic acid moiety that confers a strong affinity with cannabinoid receptors.^18^ The two most extensively studied endocannabinoids are arachidonoyl ethanolamide (AEA) and 2-arachidonoyl glycerol (2-AG).^18^

The components and signaling pathways of the endocannabinoid system are similar in most organs throughout the body.^18^ As endogenous or exogenous cannabinoids arrive at target cells, both CB1R and CB2R are stimulated with heterotrimeric G-proteins and suppress adenylate cyclase to inhibit the phosphorylation of protein kinase A. In contrast, mitogen-activated protein kinase is stimulated to regulate additional gene expressions.^14^,^18^ In the case of CB1R, when heterodimeric G-protein is stimulated, it directly inhibits the membrane’s calcium channels and stimulates the potassium channels to inhibit the release of neurotransmitters in neuronal cells.^14^ However, the activation of cannabinoid receptor–mediated signaling pathways may differ depending on the type of cells stimulated.^18^

### Endocannabinoid Production and Degradation

**Figure 1:**
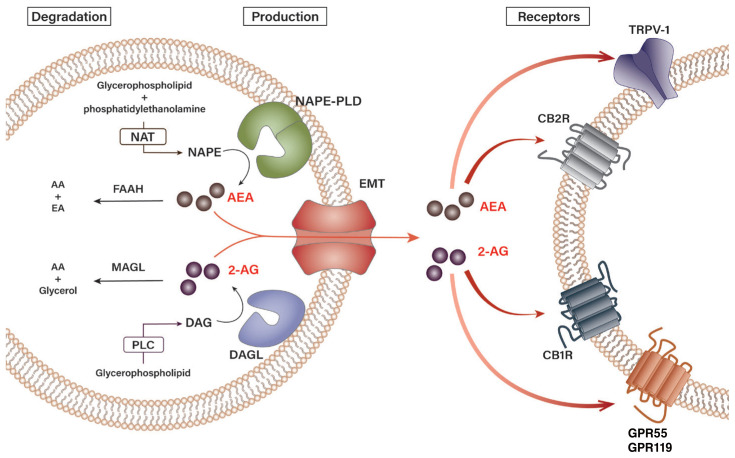
Biosynthesis and degradation pathways of endocannabinoids. Endogenous cannabinoids (endocannabinoids)—arachidonoyl ethanolamide (AEA) and 2-arachidonoyl glycerol (2-AG)—have distinct pathways of synthesis and degradation in cells. N-arachidonoyl-phosphatidylethanolamine (NAPE) is synthesized from glycerophospholipid and phosphatidylethanolamine by N-acyltransferase (NAT). Upon stimulation, NAPE subsequently gets hydrolyzed by NAPE-specific phospholipase D (NAPE-PLD) to produce AEA. Synthesis of 2-AG begins with the production of *sn*-1-acyl-2-arachidonoyl-glycerol (DAG) from glycerophospholipid by phospholipase C (PLC), which is then hydrolyzed by diacylglycerol lipase (DAGL) to 2-AG. The synthesized AEA and 2-AG are transported out of the cell by an endocannabinoid membrane transporter (EMT). The released AEA and 2-AG then bind their cannabinoid and noncannabinoid receptors in the neighboring cells to transduce extracellular signals. 2-AG binds both cannabinoid-1 receptor (CB1R) and cannabinoid-2 receptor (CB2R) with similar affinity, whereas AEA has a stronger affinity for CB1R. 2-AG and AEA also bind transient receptor potential vanilloid type-1 (TRPV-1) and orphan G protein-coupled receptors 55 (GPR55) and 119 (GPR119). AEA is hydrolyzed into arachidonic acid (AA) and ethanolamine (EA) by fatty acid amide hydrolase type-1 (FAAH-1) and type-2 (FAAH-2), and N-acylethanolamine-hydrolyzing acid amidase (NAAA), whereas 2-AG is degraded into AA and glycerol by monoacylglycerol lipase (MAGL) and FAAH.

Endocannabinoids are biosynthesized through various pathways from several precursors of phospholipids located in the cellular membrane. Figure 1 schematically summarizes the biosynthesis and degradation pathways of endocannabinoids AEA and 2-AG.^11^,^14^,^19^ N-arachidonoyl-phosphatidylethanolamine (NAPE), a phospholipid precursor located in the cell membrane, is preferentially synthesized from glycerophospholipid and phosphatidylethanolamine by N*-*acyltransferase (NAT) and sequentially hydrolyzed by the NAPE-specific phospholipase D (NAPE-PLD) in response to stimulation, subsequently resulting in the production of AEA (see Figure 1).^19^ Degradation of AEA involves its hydrolysis into arachidonic acid and ethanolamine by a number of enzymes, namely fatty acid amide hydrolase (FAAH) and N-acylethanolamine-hydrolyzing acid amidase (NAAA), in the intracellular space.^20^,^21^ As for 2-AG, *sn*-1-acyl-2-arachidonoyl-glycerol (DAG) is first produced from the intracellular glycerophospholipid by phospholipase C at the plasma membrane. Then, DAG is subsequently hydrolyzed by diacylglycerol lipase (DAGL) to 2-AG.^22^ Although the chemical structures of DAGL-alpha and DAGL-beta are slightly different, their preference for ligands is similar.^14^ Interestingly, a study has shown that DAGL-alpha has a more dominant role over DAGL-beta in regulating the levels of 2-AG in the brain, but the opposite was observed in the liver. In fact, only DAGL-beta, but not DAGL-alpha, has been reported to be expressed in HSCs of fatty mouse liver.^7^,^10^ Unlike AEA, 2-AG is believed to be degraded into arachidonic acid and glycerol by several enzymes, FAAH, and monoacylglycerol lipase (MAGL).^22^

Generally, the activation of both NAPE-PLD and DAGL is triggered by changes in the intracellular calcium signaling.^12^,^20^ When calcium influx occurs in a cell by a specific stimulus, the intracellular concentration of AEA or 2-AG increases due to the activation of endocannabinoid-producing enzymes. The newly synthesized endocannabinoids are then transported from the cytoplasm out of the cell by a specific transporter, the endocannabinoid membrane transporter.^11^,^21^ Because of their hydrophobic properties, the released endocannabinoids have high binding affinities to the membrane, enabling them to rapidly bind to their specific receptors and induce biological responses in the neighboring cells. For instance, the AEA and 2-AG generated by the activation of endocannabinoid-producing enzymes stimulate hepatic CB1R to induce de novo lipogenesis in nonalcoholic and alcoholic fatty liver.^7^,^23^ In general, 2-AG acts as a full agonist at these cannabinoid receptors, whereas AEA has a weaker potency as an agonist.^13^ Although levels of 2-AG and AEA in peripheral tissues vary, 2-AG (~ 0.8 pmol/mg tissue) is maintained at higher levels than AEA (~ 1.1 fmol/mg tissue) in the liver.^7^ In terms of alcohol-mediated endocannabinoid production, studies have demonstrated that chronic ethanol exposure or consumption induces 2-AG production in cerebellar granule neurons in vitro or in HSCs in vivo, respectively.^7^,^10^,^24^

### Cannabinoid Receptor Expression

In line with their differences in synthesis, AEA and 2-AG have different affinities for their respective cannabinoid receptors.^12^ AEA has a stronger affinity for CB1R than for CB2R, whereas 2-AG has a similar affinity for both CB1R and CB2R. In addition, AEA and 2-AG are also known to bind receptors other than the cannabinoid receptors, such as the transient receptor potential vanilloid type 1 (TRPV-1) and the orphan G protein-coupled receptors 55 (GPR55) and 119 (GPR119).^14^,^19^ However, with little being known, the detailed physiological effect of endocannabinoid binding to these non-cannabinoid receptors on the cellular pathophysiology in the liver remains enigmatic.

**Figure 2:**
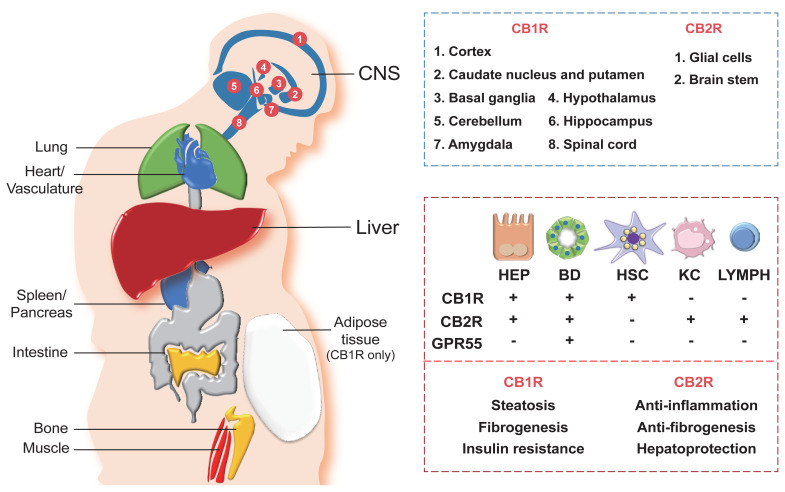
Distribution of cannabinoid receptors in various organs and hepatic cells. Cannabinoid receptors, cannabinoid-1 receptor (CB1R) and cannabinoid-2 receptor (CB2R), are expressed in various central and peripheral organs. CB1R and CB2R are most abundantly expressed in the central nervous system (CNS), where different parts of the CNS express either CB1R or CB2R (blue box). Both CB1R and CB2R are also expressed in peripheral organs including the heart, lung, spleen, pancreas, intestine, bone, muscle, and liver, as well as in the vascular system. Adipose tissues only express CB1R. In the liver, diverse types of cells—including hepatocytes (HEP), cholangiocytes (bile duct [BD] epithelial cells), hepatic stellate cells (HSC), Kupffer cells (KC), and lymphocytes (LYMPH)—differentially express cannabinoid receptors (CB1R and CB2R) and orphan G protein-coupled receptor 55 (GPR55), a noncannabinoid receptor that binds with endocannabinoids 2-AG and AEA (red box, top). Different functions of CB1R and CB2R in the liver are also indicated (red box, bottom).

Once the endocannabinoids, either synthetic or endogenous, bind to their cannabinoid receptors, both the CB1R and CB2R get stimulated enough to rapidly transduce extracellular signals into cells.^25^, ^26^ With regards to their expression, they are widely distributed throughout our body as summarized in Figure 2. CB1R is predominantly distributed in the central and peripheral nervous system, including the sensorial peripheral and sympathetic nerves in humans and mice.^26^ However, abundant evidence has confirmed that CB1R is also characteristically expressed in several peripheral tissues and organs, including liver, lung, gastrointestinal tract, urinary tract, thyroid, pancreas, heart, vascular endothelium, adipose tissue, reproductive organs, skeletal muscles, and immune system (see Figure 2).^11^,^25^ Unlike CB1R, CB2R is mainly expressed in cells and organs that are responsible for controlling peripheral hematopoiesis or immune functions (see Figure 2).^25^,^26^ For example, macrophages, neutrophils, monocytes, B lymphocytes, T lymphocytes, and microglial cells are representative of CB2R-expressing cells. Recently, an increasing number of reports have expanded the scope of peripheral tissue known to contain CB2R to include skin nerve fibers, keratinocytes, bone cells (i.e., osteoblasts, osteocytes, and osteoclasts), and somatostatin-secreting cells in the pancreas.^27^

### Cannabinoid Receptor Activation in the Liver

Early research on endocannabinoids focused on demonstrating the mechanism of psychoactive symptoms and their neurologic signals caused by the stimulation of CB1R in the brain.^13^,^26^ However, little attention was paid to the biological roles of the hepatic endocannabinoid system despite the discovery of cannabinoid receptors in the liver.^9^ Nowadays, emerging lines of evidence have shown that diverse types of the hepatic cells not only express CB1R or CB2R but also employ them in the hepatic pathophysiology, drawing attention to the critical correlation between chronic liver diseases and cannabinoid receptor signaling.^28^

Hepatocytes, the parenchymal cells of the liver, mainly express CB1R, but the level of expression is relatively low in the homeostatic condition (see Figure 2). However, CB1R expression is tremendously elevated in pathological conditions, such as alcoholic and nonalcoholic steatosis, primary biliary cirrhosis, and hepatocellular carcinoma.^9^,^19^,^29^ CB2R is rarely expressed in the steady state of the liver, but its expression is elevated in immune cells during the occurrence of hepatic regeneration and diseases such as NAFLD, fibrosis, and hepatocellular carcinoma.^29^,^30^ As opposed to the hepatocytes, the cannabinoid signaling in hepatic nonparenchymal cells is relatively less explored. CB1R expression in HSCs was shown to have increased significantly in the rodent fibrosis model and cirrhotic human liver,^11^,^21^ suggesting that endocannabinoids can act as pro-fibrogenic mediators in the liver. Moreover, the authors’ previous studies have demonstrated that alcoholic steatosis is exacerbated through CB1R activation in hepatocytes by 2-AG produced from HSCs.^7^,^10^ CB1R is also expressed in cholangiocytes, or bile duct epithelial cells, which are related to the pathophysiology of liver cirrhosis and primary biliary cirrhosis.^31^ Furthermore, several studies have identified the close association of CB2R expressions in hepatic nonparenchymal cells and NAFLD progression, but detailed mechanisms have yet to be investigated. The distribution of the cannabinoid receptors in hepatic cells is briefly described in Figure 2.

## Cannabinoid Signaling in the Pathogenesis of ALD

### Alcohol Exposure and the Endocannabinoid System in ALD

Because alcohol exposure is considered a critical factor in causing complex physiological or pathological changes in the endocannabinoid system, curiosity about the biological function of cannabinoid receptors in ALD began to arise.^9^,^28^ Consequently, the endocannabinoid system and its receptors were found to be involved in the pathophysiological mechanisms of ALD by regulating immune function, metabolic modulation, and inflammatory response in the onset and progression of ALD.^29^, ^32^ Because the expression of CB1R and CB2R is well identified in hepatocytes and various nonparenchymal cells in the liver, accurate comprehension of the regulatory mechanisms by which alcohol exposure generates or stimulates the production of endocannabinoids—as well as the effects of alcohol on the activation of cannabinoid receptors—could lead to a breakthrough in understanding the exact pathophysiology of ALD and in discovering potential therapeutic targets.

### Alcoholic Liver Injury Through Cannabinoid Signaling

The pathological changes in the endocannabinoid system can lead to the development of several chronic liver diseases. Because the expressions of CB1R and CB2R increase in pathological conditions such as NAFLD, primary biliary cirrhosis, liver cirrhosis, and hepatocellular carcinoma, the hepatic endocannabinoid system is most likely to affect the onset of ALD.^9^,^28^,^29^

**Figure 3:**
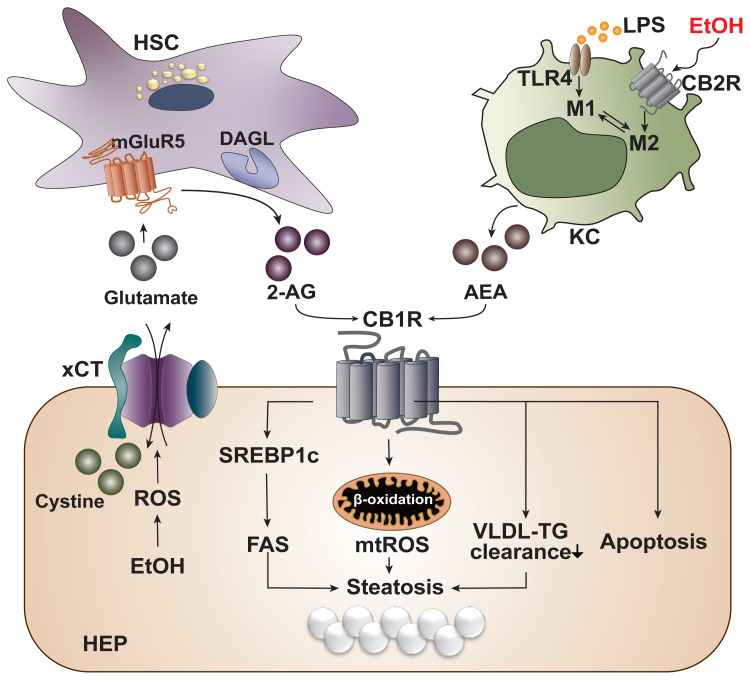
Cannabinoid signaling in the pathogenesis of alcohol-associated liver disease. Alcohol is mainly metabolized in hepatocytes (HEP) of the liver during which reactive oxygen species (ROS) is generated as a cellular stress response. The generated ROS stimulates and activates a cystine/glutamate antiporter (xCT) for the influx of cystine in exchange for the efflux of glutamate. The excreted glutamate then binds to a metabotropic glutamate receptor 5 (mGluR5) expressed in the neighboring hepatic stellate cells (HSC), inducing the production of 2-arachidonoyl glycerol (2-AG) by diacylglycerol lipase (DAGL). 2-AG produced in the HSC binds to cannabinoid-1 receptors (CB1R) expressed in the plasma membrane of neighboring HEP to induce de novo lipogenesis via the upregulation of sterol regulatory element-binding protein 1c (SREBP1c) and fatty acid synthase (FAS). This forms a bidirectional paracrine loop pathway through which HEP and HSC in close proximity can metabolically regulate each other. Activation of CB1R can also induce β-oxidation of fatty acids in mitochondria, generating mitochondrial ROS (mtROS), which ultimately contributes to the accumulation of fat, or steatosis. Activated CB1R perturbs the excretion of triglyceride (TG) in the form of TG-rich very low-density lipoprotein (VLDL), further contributing to hepatic steatosis. CB1R activation is also known to induce apoptosis of cells. Kupffer cells (KC) normally become activated via the lipopolysaccharide (LPS)/toll-like receptor 4 (TLR4) stimulation and acquire a pro-inflammatory (M1) phenotype. However, when the CB2R expressed in Kupffer cells are stimulated by ethanol, they obtain an anti-inflammatory (M2) phenotype. Activated Kupffer cells then produce arachidonoyl ethanolamide (AEA), which also binds and activates CB1R in the neighboring HEP.

With the liver as the principal organ of alcohol metabolism, the majority of the alcohol consumed enters the liver to be metabolized, consequently activating the stress responses such as the production of ROS, inflammatory cytokines, or endoplasmic reticulum stress. These responses result in reduced fatty acid oxidation and enhanced hepatic lipogenesis.^6^ Several animal experiments have established that chronic alcohol consumption could exacerbate alcoholic fatty liver by triggering abnormal CB1R-mediated signaling.^7^,^10^ However, the authors’ recent studies have clearly demonstrated that chronic alcohol consumption induces oxidative stress-mediated glutamate excretion from hepatocytes, which triggers the activation of mGluR5 to produce 2-AG, but not AEA, in HSCs via DAGL-beta. This, in turn, stimulates paracrine activation of hepatic CB1R,^7^,^10^ which leads to the subsequent elevation of the expression of sterol regulatory element-binding protein-1c (SREBP1c), a representative lipogenic transcription factor located downstream of the CB1R signaling pathway.^7^,^30^ As a result, the expression of target proteins of SREBP1c—namely acetyl coenzyme A (CoA) carboxylase and fatty acid synthase—are elevated, thereby inducing de novo lipogenesis in hepatocytes (see Figure 3).^23^,^33^ This study served as a crucial opportunity to identify the involvement of the endocannabinoid system in metabolic regulation through bidirectional interaction between hepatocytes and HSCs in the liver. The fatty acids produced are then converted into triglyceride (TG), which should be excreted from the liver in the form of TG-rich very-low-density lipoprotein (VLDL). However, pharmacological blockade of CB1R (AM6545 and rimonabant) decreases the hepatocytes’ ability to clear TG-rich VLDL, significantly reducing hepatic TG levels and markedly increasing the release of TG-rich VLDL in alcoholic and nonalcoholic fatty liver.^7^,^34^

In alcoholic liver injury and inflammation, the various types of ROS are one of the most important influential factors in the progression of ALD. The ROS is mainly generated through two metabolizing pathways that utilize different enzymes or proteins: alcohol dehydrogenase and cytochrome P450 2E1 (CYP2E1), which is a membrane protein that forms the cytochrome P450-dependent microsomal ethanol oxidizing system.^6^ The importance of ROS in alcoholic liver injury has been portrayed in a study that reported the close relationship between the endocannabinoid system and ROS-induced liver injury in the pathophysiology of chronic alcohol consumption.^35^ In this study, ethanol-induced 2-AG preferentially induced CB1R activation, followed by an upregulation in gene expression of estrogen-related receptor gamma (ERR-gamma), an orphan nuclear receptor. The authors explained that the increased expression of ERR-gamma enhances CYP2E1 induction, resulting in ROS-induced alcoholic liver injury. In addition, when ethanol was fed chronically to CB1R knockout mice, the expression of ERR-gamma and CYP2E1 decreased and alcoholic liver injury was significantly attenuated. Furthermore, administration of GSK5182, which is a selective inverse agonist of ERR-gamma, ameliorated alcoholic liver injury by reducing oxidative stress, confirming the criticality of cannabinoid receptor signaling in ROS-induced alcoholic liver injury.^35^ Among the various inflammatory pathways activated in ALD, Kupffer cells, which are macrophages that reside in liver tissue, execute a crucial role in the onset of hepatic inflammation.^6^ Currently, the most well-known mechanism of Kupffer cell activation is via lipopolysaccharide (LPS)/toll-like receptor 4 (TLR4) stimulation, by which the Kupffer cells acquire a pro-inflammatory phenotype.^6^ Like other cells in the immune system, Kupffer cells mainly express CB2R rather than CB1R, and activation of CB2R exerts an anti-inflammatory property on Kupffer cells in the development of ALD.^29^ In fact, when wild-type mice were fed with alcohol, Kupffer cells were polarized to the anti-inflammatory (M2) phenotype, whereas the pro-inflammatory (M1) phenotype was amplified in CB2R-deficient Kupffer cells in response to LPS stimulation.^36^ In line with this observation, Kupffer cells also have been shown to acquire a protective property via the activation of their CB2R as regulated by an autophagy-dependent pathway, which further supports the essential role of CB2R in Kupffer cells.^37^ Moreover, chronic alcohol consumption instigates the disruption of the intestinal epithelium, causing changes in gut permeability and increasing the level of LPS in the hepatic portal flow. Consequently, Kupffer cells become activated by TLR4. A study by Szabady et al. suggested a conceivable interplay between intestinal endocannabinoids and ALD. The authors demonstrated that intestinal endocannabinoids produced by epithelial cells could prevent inflammation and maintain homeostasis in a healthy gut by modulating neutrophil influx.^38^ Thus, intestinal endocannabinoids might play beneficial roles in ALD-mediated gut leakage and the subsequent translocation of LPS to the liver.

CB1R also was found to modulate alcohol-induced liver fibrosis.^39^ A study conducted by Patsenker et al. observed a strong expression of CB1R in the fibrotic septa of patients with alcohol-associated liver cirrhosis, and genetic and pharmacologic inhibition of CB1R attenuated both the hepatic inflammation and the alcoholic liver fibrosis by suppressing HSC activation.^39^ Although it is well established that CB1R is involved in the development of hepatic steatosis and fibrosis, relatively few studies have examined the role of CB2R in the pathophysiology of ALD. In a comparison study for the severity of hepatic steatosis, inflammation, and fibrosis using CB1R and CB2R knockout mice, the CB2R knockout mice showed severe fibrosis with aggravated steatosis and inflammation compared to those of the wild-type and CB1R knockout mice. This observation could be explained by the fact that the collagen production in activated HSCs was amplified in CB2R knockout mice,^40^ indicating the protective role of CB2R in the progression of alcoholic liver fibrosis.

In brief, endocannabinoids have been found to have diverse effects on the pathophysiology of chronic liver disease, and various in vivo and in vitro experiments have been performed to investigate the characteristics of CB1R and CB2R in different types of ALD. To date, it is known that CB1R activation aggravates inflammation, steatosis, and fibrosis through the reduction of fatty acid oxidation and TG-VLDL secretion, enhanced de novo lipogenesis, and activation of HSCs, whereas CB2R inhibits inflammation and steatosis and has anti-fibrotic properties by exerting anti-inflammatory functions on Kupffer cells.^29^,^32^ Figure 3 summarizes the opposite roles of CB1R and CB2R in the progression of ALD.

### Glutamate-Mediated Endocannabinoid Production

As described earlier, one of the key mechanisms underlying the development of alcoholic fatty liver is the CB1R-mediated de novo lipogenesis in hepatocytes via the metabolic loop pathway.^7^ However, questions remain as to which metabolic triggers lead to increased production of 2-AG in HSCs. Recently, the authors of this review substantiated that oxidative stress mediates the excretion of glutamate from the hepatocyte, stimulating the activation of mGluR5, which binds to glutamate, in nearby HSCs and leading to increased 2-AG production (see Figure 3).^10^ Similar to other reports, this report also found that chronic alcohol consumption depleted antioxidant glutathione through the inhibition of the methionine cycle and the transsulfuration system, resulting in a shortage of cysteine. However, this study had a more striking discovery. First, the CYP2E1-mediated ROS production in hepatocytes significantly increased the xCT (cystine/glutamate antiporter)-mediated uptake of extracellular cystine, in exchange for the excretion of cytosolic glutamate, to compensate for the glutathione deficiency. Second, this parallel release of glutamate stimulated activation of mGluR5 in HSCs, which led to the production of 2-AG through mediation by DAGL-beta. As a result, the 2-AG produced activated CB1R in neighboring hepatocytes, inducing de novo lipogenesis. These findings suggest a bidirectional paracrine loop between hepatocytes and HSCs, named the “metabolic loop pathway,” where both hepatocytes and HSCs regulate each other by either producing a neurotransmitter or expressing its receptor. Thus, the authors proposed a novel view of concept through this bidirectional signaling that utilizes a neurotransmitter, an endocannabinoid, and their respective receptors to operate at a metabolic synapse between hepatocytes and HSCs. In vivo experiments using genetic or pharmacologic inhibition of xCT or mGluR5 showed an improvement in alcohol-induced hepatic steatosis. More interestingly, plasma levels of glutamate were found to be elevated in ALD patients with hepatic steatosis and hepatitis but not in patients with fibrosis and cirrhosis, which suggests that the function of glutamate is not limited to the hepatic steatosis and further studies are strongly required to address this curiosity. In summary, the discovery of a bidirectional loop pathway between hepatocytes and HSCs suggested a new mechanism for the development of ALD, proposing the possibility of its application as a novel pharmacological target or an opportunity for glutamate as a prospective diagnostic marker in ALD.

## Therapeutic Implications for ALD

### Past and Current Pharmacological Approaches

Various animal experiments have established that hepatic endocannabinoids and their receptors play fundamental roles in the pathophysiology of chronic liver diseases, and pharmacological targeting of CB1R and CB2R for the treatment of liver diseases has been attempted.^29^ Table 1 summarizes the effects of cannabinoid receptor–modulating drugs and their targets in animal models of ALD to date. Unfortunately, most clinical trials have been performed on patients with obesity, metabolic syndrome, and NAFLD, and only a few studies have explored and reported the beneficial efficacies of CB1R antagonists in the progression of hepatic steatosis, inflammation, and fibrosis.^21^,^25^ In fact, clinical trials of cannabinoid receptor inhibitors have not been carried out in patients with ALD owing to the side effects of the drugs. For example, in a meta-analysis of nine clinical trials, adverse events, such as depression, anxiety, and nausea, were commonly observed with rimonabant at a dose of 20 mg per day for 6 to 24 months even though it had clinically meaningful results in metabolic disorders.^41^

Recently, a chemical compound that acts as a peripherally restricted antagonist of CB1R has been developed, which showed negligible CNS penetration and remarkable attenuation of alcoholic steatosis in mice.^42^ Thus, there is a silver lining in the possibility that with refinement and adjustment, this chemical might be a profound lead compound that could undergo clinical trials as a novel therapeutic target. In short, a growing number of experimental findings on the involvement of hepatic endocannabinoids in the pathophysiology of ALD has enabled the development of endocannabinoid-based or cannabinoid receptor–based pharmacological approaches that, it is hoped, could become a novel therapeutic strategy for ALD.

### Limitation of the Current Cannabinoid-Based Treatment

Until now, there have been several clinical trials and reports in which a CB1R antagonist has been administered as treatment for obesity or metabolic risk factors.^43^–^45^ The two most notable clinical trials are the ADAGIO-Lipids Trial and the Rimonabant in Obesity (RIO)-Europe study. In these clinical trials, cardiometabolic risk markers, such as body weight and lipid profiles, improved significantly when rimonabant, a well-known CB1R-selective antagonist, was administered to obese patients for 1 or 2 years, but the treatments were discontinued because of the psychiatric side effects including anxiety and depression.^45^ Since then, the development of drugs with a mode of action restricted to the endocannabinoid system in the periphery has been undertaken. For example, peripheral organ-specific CB1R inverse agonist and antagonist (i.e., JD5037 and AM6545) were developed to reduce neuropsychiatric side effects, which were successful in reducing and improving cardiometabolic risks and hepatic steatosis in animal experiments.^34^,^46^

Apart from the CB1R antagonist, the pharmacological potential of the CB2R agonist, which is known to have hepatoprotective effects, also has been reevaluated.^36^ Although only observed in mice, a study has confirmed that administration of JWH-133 (a CB2R agonist) exhibited improved alcoholic liver injury in mice by inducing the polarization of Kupffer cells into an M2 phenotype.^36^,^37^ Interestingly, according to a recent cross-sectional study, cannabis users showed a significantly reduced prevalence of ALD of all spectrums (alcoholic steatosis, alcoholic steatohepatitis, alcohol-associated cirrhosis, and hepatocellular carcinoma). However, the underlying mechanism remains in question.^47^ Based on the description above, one could speculate that the cannabis absorbed might activate CB2 receptors in immune cells or prevent intestinal leakage of endotoxins including LPS. Therefore, to date, no drugs targeting the endocannabinoid system are available for direct application to clinical trials in ALD patients, and further studies are required to study underlying mechanisms and to develop a treatment specifically effective for ALD.

## Conclusions

Endocannabinoids are membranous lipid mediators that regulate diverse physiological functions in both the CNS and the peripheral organs, including the liver. Over the past 30 years, it has been found that the endocannabinoid system is involved in a variety of pathways associated with the onset, or the progression, of several diseases, including ALD. The endocannabinoid system has been observed in both the hepatocytes and various nonparenchymal cells in the liver, in which the endocannabinoid production and its receptor activation may contribute to the development of a spectrum of ALD, ranging from simple alcoholic steatosis to more severe forms such as steatohepatitis and fibrosis. Therefore, understanding the precise physiology of the endocannabinoid system in the liver and unveiling the mechanism underlying the association between ALD progression and hepatic endocannabinoid signaling seem to bear a paramount significance for the advancement of ALD treatment, as well as for the treatment of other chronic liver diseases (e.g., NAFLD, viral hepatitis). Moreover, developing efficacious and highly selective cannabinoid receptor–modulating drugs could be a major breakthrough in the treatment of ALD.

However, efforts to develop second- and third-generation CB1R antagonists must overcome the complications caused by the first generation of CB1R antagonists, which were able to penetrate the blood-brain barrier and produced critical psychiatric side effects. Furthermore, careful implication of the combinatorial effects of CB1R antagonist and CB2R agonist may bring about promising outcomes for the treatment of ALD in the future.

## Figures and Tables

**Table 1:** Effects of Various Cannabinoid Receptor–Modulating Drugs and Their Target Cells in Different Animal Models of Alcohol-Associated Liver Disease, by Pharmacological Trial

Trial	Reagent	Receptor	Target Cell	Action	Research Model	Effect and Results
Jeong et al. (2008)^7^	Rimonabant	CB1R	Hepatocyte	Antagonist	Alcoholic fatty liver	Reduce steatosis (Lipogenesis↓, fatty acid oxidation↑)
Patsenker et al. (2016)^19^	Rimonabant	CB1R	HSC	Antagonist	In vitro experiment	Induce apoptosis Reduce pro-fibrotic property
Louvet et al. (2011)^36^	JWH-133	CB2R	Kupffer cell	Agonist	Alcoholic fatty liver	M2 polarization of Kupffer cell (Steatosis↓, inflammation↓)
Kim et al. (2013)^35^	GSK5182	ERR-gamma	Hepatocyte	Antagonist	Alcoholic fatty liver and inflammation	Reduce oxidative stress (CYP2E1 expression↓, hepatocyte apoptosis↓)
Amato et al. (2018)^42^	Compounds 25	CB1R	Hepatocyte	Antagonist	Alcoholic fatty liver	Peripherally restricted purine antagonist
Choi et al. (2019)^10^	CTEP	mGluR5	HSC	Antagonist	Alcoholic fatty liver	Inhibit mGluR5 and reduce steatosis (Lipogenesis↓, CB1R expression↓)
Choi et al. (2019)^10^	Sulfasalazine	xCT	Hepatocyte	Antagonist	Alcoholic fatty liver	Inhibit xCT and reduce steatosis (Lipogenesis↓, CB1R expression↓)

*Note:* The upward arrow (↑) indicates an increase, and the downward arrow (↓) indicates a decrease. CB1R, cannabinoid-1 receptor; CB2R, cannabinoid-2 receptor; CYP2E1, cytochrome P450 family 2 subfamily E member 1; ERR-gamma, estrogen-related receptor-gamma; HSC, hepatic stellate cell; mGluR5, metabotropic glutamate receptor 5; xCT, cystine/glutamate antiporter.
